# Normobaric hyperoxia plays a neuroprotective role after cerebral ischemia by maintaining the redox homeostasis and the level of connexin43 in astrocytes

**DOI:** 10.1111/cns.13875

**Published:** 2022-06-14

**Authors:** Zhifeng Qi, Shuhua Yuan, Ke Jian Liu, Xunming Ji

**Affiliations:** ^1^ Department of Neurology, Beijing Institute for Brain Disorders Xuanwu Hospital of Capital Medical University Beijing China; ^2^ Department of Pharmaceutical Sciences University of New Mexico Albuquerque New Mexico USA; ^3^ Center of Stroke, Beijing Institute for Brain Disorders Capital Medical University Beijing China

**Keywords:** acute ischemic stroke, astrocyte, gap junction connexin43, glutathione peroxidase 4 (GPX4), normobaric hyperoxia, oxidative stress

## Abstract

**Introduction:**

Acute cerebral ischemia is caused by an insufficient blood supply to brain tissue. Oxygen therapy, which is able to aid diffusion to reach the ischemic region, has been regarded as a possible treatment for cerebral ischemia. Recent animal and pilot clinical studies have reported that normobaric hyperoxia (NBO) showed neuroprotective effects if started soon after the onset of stroke. However, little is known about the role and mechanism of NBO treatment in astrocytes. Connexin43, one of the main gap junction proteins in astrocytes, is extremely sensitive to hypoxia and oxidative stress after cerebral ischemia.

**Aims:**

In the present study, we used sutures to develop an ischemia/reperfusion model in rats to mimic clinical recanalization and investigated the role of connexin43 in NBO‐treated stroke rats, as well as the underlying mechanism of NBO therapy.

**Results:**

Normobaric hyperoxia treatment maintained the homeostasis of oxidoreductases: glutathione peroxidase 4 (GPX4) and NADPH oxidase 4 (two important oxidoreductases) and rescued the ischemia/reperfusion‐induced downregulation of connexin43 protein in astrocytes. Furthermore, NBO treatment attenuated cerebral ischemia‐induced cytochrome c release from mitochondria and was involved in neuroprotective effects by regulating the GPX4 and connexin43 pathway, using Ferrostatin‐1 (an activator of GPX4) or Gap27 (an inhibitor of connexin43).

**Conclusions:**

This study showed the neuroprotective effects of NBO treatment by reducing oxidative stress and maintaining the level of connexin43 in astrocytes, which could be used for the clinical treatment of ischemic stroke.

## Graphical abstract

Acute ischemic stroke impairs the redox balance [decreasing glutathione peroxidase 4 (GPX4) and increasing NADPH oxidase 4 (NOX4)] and reduces the connexin43 expression in astrocytes, leading to mitochondrial damages and cerebral injury. Normobaric hyperoxia (NBO) plays a neuroprotective role by reducing oxidative stress and maintaining the level of connexin43 in astrocytes.

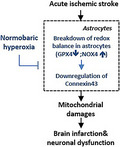

## 1 INTRODUCTION

Acute cerebral ischemia is caused by the insufficient blood supply to brain tissues. With the development and application of vascular recanalization therapies, patients in acute stages can achieve successful recanalization to remove the vascular obstruction and restore blood flow.^1^, ^2^ However, more than 50% of patients who receive recanalization do not achieve the expected good prognosis.^3^

One of the main reasons involves irreversible damage to brain tissue caused by acute ischemia. In the past, numerous treatments focusing on neuronal protection have failed in clinical trials,^4^ suggesting that other supporting cells in brain tissues should be considered, such as astrocytes, which play crucial roles in regulating and supporting neuronal activities and metabolism.

An insufficient oxygen supply is critical for the generation of acute cerebral ischemia. Because the brain is sensitive to hypoxia, oxygenation that can improve the level of tissue oxygen has been regarded as a potential treatment for ischemic stroke.^5^ Recent studies showed that a short duration of normobaric hyperoxia (NBO) during ischemia maintained the interstitial pO_2_ level in the penumbra close to the normal tissues.^6^, ^7^ Latest clinical studies demonstrated that NBO greatly reduces infarct volume and improves clinical outcomes.^8^ Recent experimental studies have reported that NBO treatment exerted neuroprotective effects, which was explained by many possible mechanisms, including suppressing oxidative stress and improving tissue metabolism.^9^

Astrocytes, the largest number of glial cells (5–10 times more than neurons) in the cortex, play important roles in supporting neuronal functions and survival during cerebral ischemia.^10^, ^11^ However, little is known about the role and mechanism of NBO therapy in astrocytes.

Connexin43, serving as a gap junction, is widely expressed in astrocytes and is responsible for the communication between cells.^12^ Studies showed that astrocytic uncoupling due to the reduction or internalization of connexin43 was very sensitive to hypoxia and that even 15‐min hypoxia resulted in 77% loss of coupling.^13^ Furthermore, a boost of free radicals deteriorated the loss of connection, impairing the communication between astrocytes and neurons. Therefore, we hypothesized that NBO may play a neuroprotective role by reducing oxidative stress and maintaining the level of connexin43 in astrocytes.

In this study, we, therefore, used sutures to develop an ischemia/reperfusion model of rats to mimic clinical recanalization and to investigate the role of connexin43 in NBO‐treated rats. We also attempted to identify the underlying mechanism of NBO therapy in astrocytes during cerebral ischemic stroke.

## 2 METHODS

### 2.1 Animal model

**FIGURE 1:**
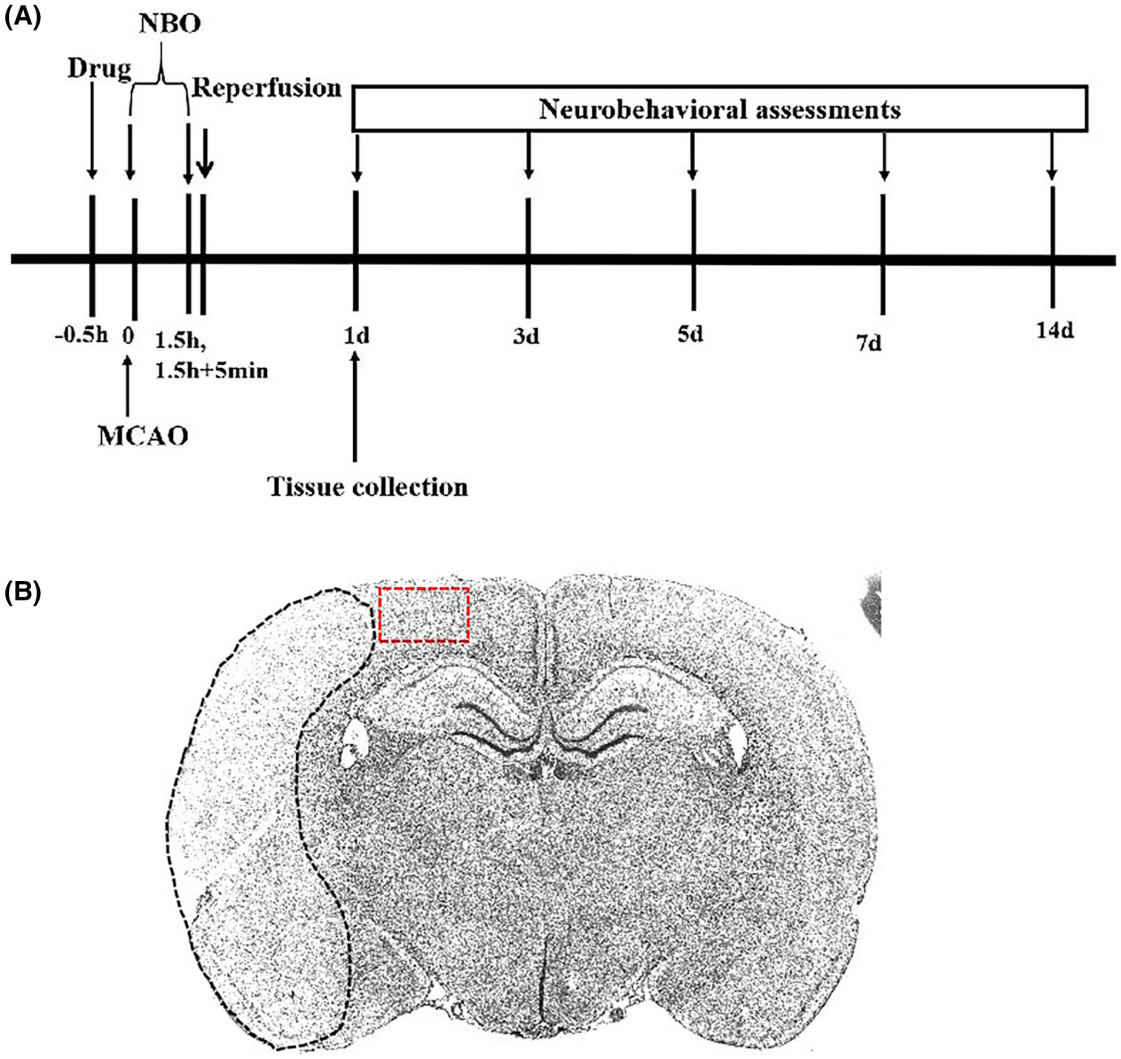
Experimental design. (A) The chart shows the experimental design including drug administration, MCAO surgery, NBO treatment, tissue collection, and neurobehavioral assessments. MCAO, middle cerebral artery occlusion; NBO, normobaric hyperoxia. (B) The schematic diagram of the focusing area (context in peri‐ischemic region, red‐dashed box) collected for western blotting or observed for immunohistochemistry. The ischemic region is outlined with the black‐dashed line

The experiments were approved by the Institutional Animal Care and Use Committee of Xuanwu Hospital of Capital Medical University (Beijing, China) and performed following the Animal Research: Reporting In Vivo Experiments (ARRIVE) 2.0 guidelines.^14^ Surgery of the middle cerebral artery (MCA) occlusion (MCAO) was performed on Sprague–Dawley male rats (295–310 g), as previously described.^15^ The experimental flowchart is shown in Figure 1A. Briefly, animals were anesthetized with 2% isoflurane. The occlusion of MCA (right) was induced using sutures (Doccol). After 90 min occlusion, the suture was carefully withdrawn for reperfusion. The MCAO model was considered successful if rats circled to the contralateral side (left), and the results were further confirmed using infarction measurements (see Infarct Measurements below). Animals in the sham group were treated with the same protocol except for placing sutures.

### 2.2 NBO treatment

Normobaric hyperoxia therapy was conducted according to our previous study.^16^ In brief, after recovery from anesthesia (generally 10 min after the onset of occlusion), the rats were immediately exposed to 100% oxygen in a box with an inlet and outlet at 5 L/min for 90 min. Similar treatment was given to the normoxic rats except for air inhalation (21% O_2_, 5 L/min).

### 2.3 Gap27 and Ferrostatin‐1 injection

Gap27 (a specific inhibitor of connexin43; 20 μg/kg; MedChemExpress) or Ferrostatin‐1 (an activator of GPX4; 125 μg/kg; MedChemExpress) was dissolved in physiological saline and then injected through the tail vein, 30 min prior to MCAO onset. The in vivo dose of Gap27 or Ferrostatin‐1 was according to a previous study of myocardial infarction^17^ and was tested in a preliminary study. Physiological saline was used in the control rats.

### 2.4 Tissue collection

After transcardiac perfusion, the brains were removed and sectioned into four slices (2‐mm thick) according to our previous study.^9^ The first and third slices were collected for western blotting or mitochondria isolation. The second slice was prepared for infarct measurements. The fourth slice was fixed for immunohistochemistry staining. Cortex tissues in the peri‐ischemic region were collected for western blotting or observed for immunohistochemistry staining (Figure 1B).

### 2.5 Infarct measurements

The infarct volume was measured using 2,3,5‐triphenyltetrazolium chloride (TTC; Sigma‐Aldrich) staining^18^ and analyzed using ImageJ software (National Institutes of Health).

### 2.6 Neurological function measurements

Neurological deficits and body weights were measured in a double‐blinded manner at 1, 3, 5, 7 and 14 days after reperfusion using the foot‐fault test, adhesive removal test, and mNSS scores, according to previous studies.^19^, ^20^, ^21^, ^22^

### 2.7 Mitochondria isolation from brain tissue

To investigate mitochondrial damage, the cytosol and mitochondrial fractions were separated as described in a previous study,^9^ using a mitochondria isolation kit (Qiagen). The intact mitochondria and cytosol fractions were stored at −80°C for further analysis.

### 2.8 Western blotting

Cortex tissues in the peri‐ischemic region were collected for western blotting (Figure 1B). Brain tissues were incubated in RIPA lysis buffer for SDS‐PAGE, as previously described.^23^ Primary antibodies were purchased from Servicebio. The level of β‐actin or Cox‐IV was used as loading controls for the cytosol and mitochondrial fractions. Unedited gel images was shown in Supplementary Information.

### 2.9 Analysis of cytochrome c release

The levels of cytochrome c (CytC) in isolated cytosolic and mitochondrial fractions were analyzed for CytC release, according to our previous studies.^9^ CytC release was calculated using the ratio of CytC in the cytosol and mitochondrial fractions.

### 2.10 Immunohistochemistry staining

Brain tissues were fixed in paraformaldehyde (4%) and embedded in paraffin and sectioned in 5‐μm slices. The primary antibodies were anti‐glial fibrillary acidic protein (GFAP; Millipore), anti‐heme oxygenase‐1 (HO‐1; Servicebio), and anti‐connexin43 (Servicebio).

Neuronal cell death was measured using co‐staining of TUNEL and NeuN (a specific indicator for neurons). The fixed slices were stained using a Click‐iT TUNEL Imaging Assay kit (Thermo Fisher Scientific) and NeuN (Thermo Fisher Scientific), according to the instructions. The 4′,6‐diamidino‐2‐phenylindole (DAPI) was used as a nuclear specific marker.

The signals in the cortex in the peri‐ischemic region were observed (the focusing area is shown in Figure 1B) and images from three fields in the focusing areas were captured using a microscope (Nikon).

### 2.11 Statistical analysis

Data were statistically analyzed using SPSS statistical software for Windows, version 26.0 (SPSS). All data were tested for normality using the Kolmogorov–Smirnov test. For neurological function data, which did not exhibit a normal distribution, differences among groups with repeated measurements were analyzed using the generalized estimate equation (GEE). For other data, which were normally distributed, differences were compared using one‐way analysis of variance (ANOVA) or repeated measures ANOVAs. The post hoc least significant difference (LSD) test was used for pairwise comparisons between mean differences. A value of *p* < 0.05 was considered significant.

## 3 RESULTS

### 3.1 NBO maintains the homeostasis of oxidoreductases (GPX4/NOX4) in astrocytes during the early phase of ischemia

Glutathione peroxidase 4 (GPX4) and NADPH oxidase 4 (NOX4), two important oxidoreductases, have been reported to regulate the generation of superoxide and/or hydrogen peroxide. In this study, we investigated whether the GPX4 and/or NOX4 signaling pathways were involved in the mechanism of NBO treatment for acute cerebral ischemia.

**FIGURE 2:**
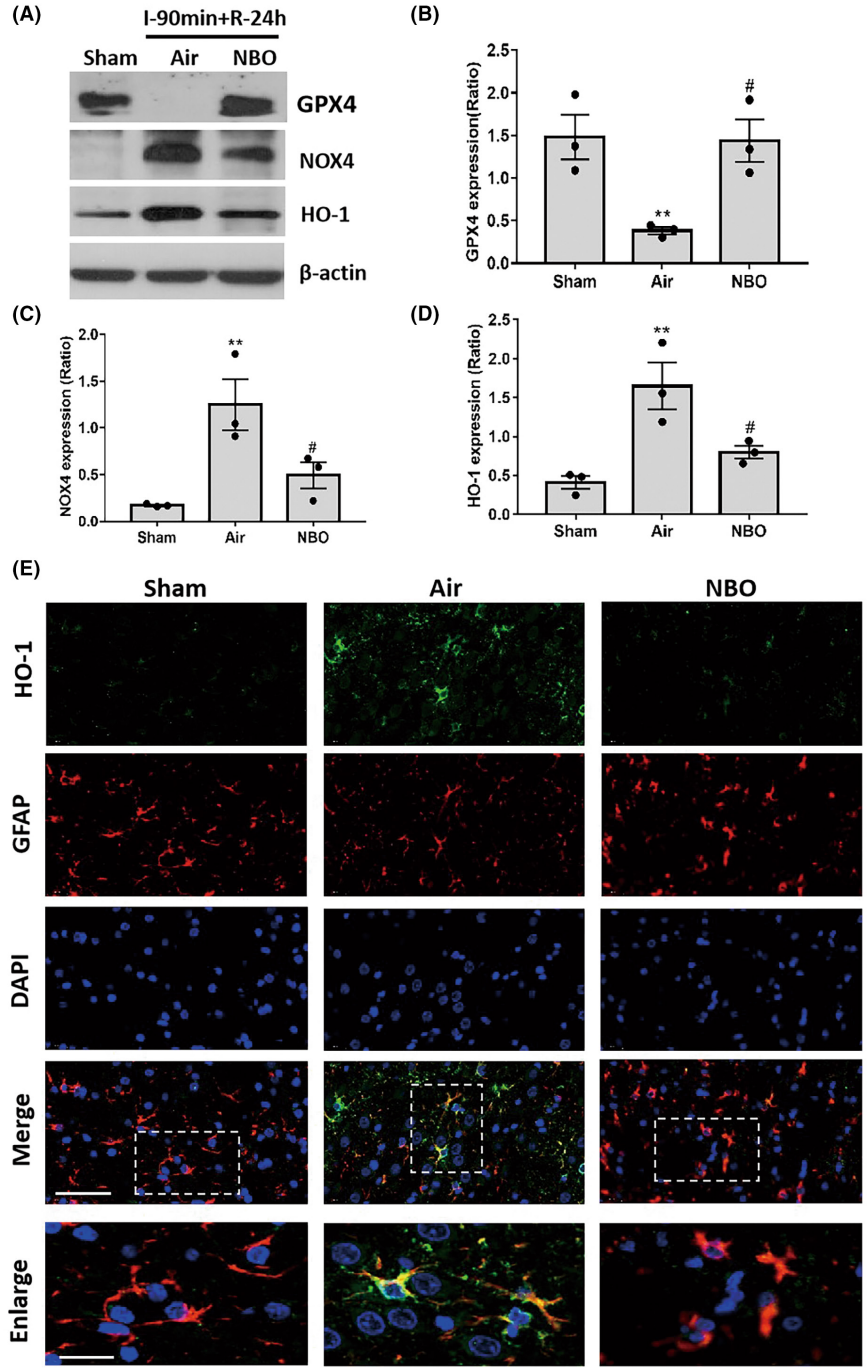
Normobaric hyperoxia maintained the homeostasis of oxidoreductases (GPX4/NOX4) in astrocytes at the early phase of ischemia. The level of GPX4, NOX4, and downstream HO‐1 was measured after 90 min ischemia plus 24 h reperfusion, using western blotting, with or without NBO treatment. (A) The representative bands of western blotting. (B–D) Quantitative analysis of expression of GPX4, NOX4, and HO‐1. Data are presented as the mean ± SEM. ^**^ *p* < 0.01, versus the sham group; ^#^ *p* < 0.05, versus the NBO group. (E) The co‐staining images of HO‐1 (green) and GFAP (red) showed the expression of HO‐1 in astrocytes with or without NBO treatment. The nucleus was stained with DAPI (blue). The images in white‐dashed boxes are enlarged. Scale bar: 50 μm (merge); 20 μm (enlarged). All data represent at least three independent experiments (three animals in each group)

The levels of GPX4 and NOX4 from ischemic tissues of NBO and normoxic rats were measured following 90‐min ischemia/24‐h reperfusion using western blotting (Figure 2A). The levels of GPX4 in normoxic rats significantly declined after 24 h ischemia/reperfusion when compared with the sham rats，whereas NBO alleviated the ischemia‐induced GPX4 decline (Figure 2B). In contrast, statistical data showed that NOX4 in intact tissues was at a very low level but increased in response to ischemia/reperfusion. NBO treatment significantly suppressed ischemia‐induced NOX4 activation (Figure 2C).

We further investigated heme oxygenase‐1 (HO‐1) expression in astrocytes, which was downstream of GPX4 and NOX4, to reflect the level of redox components in ischemic astrocytes with or without NBO treatment using western blotting (Figure 2A,D). Statistical analysis showed that HO‐1 expression was significantly increased after ischemia/reperfusion, which was suppressed in NBO‐treated rats (Figure 2D).

The expressions of HO‐1 in astrocytes in the cortex of the peri‐ischemic region were observed using immunohistochemistry. The co‐staining images showed that the HO‐1 signal was barely seen in normal tissues from the sham group. HO‐1 signals were significantly increased and mostly co‐localized in GFAP‐positive cells in response to ischemia/reperfusion, which was suppressed by NBO treatment (Figure 2E). Together, these results suggested that NBO treatment retained the redox balance in astrocytes during the early phase of ischemia/reperfusion, by using the GPX4/NOX4 pathway.

### 3.2 NBO treatment improves the expression of connexin43 in astrocytes after ischemia/ reperfusion

**FIGURE 3:**
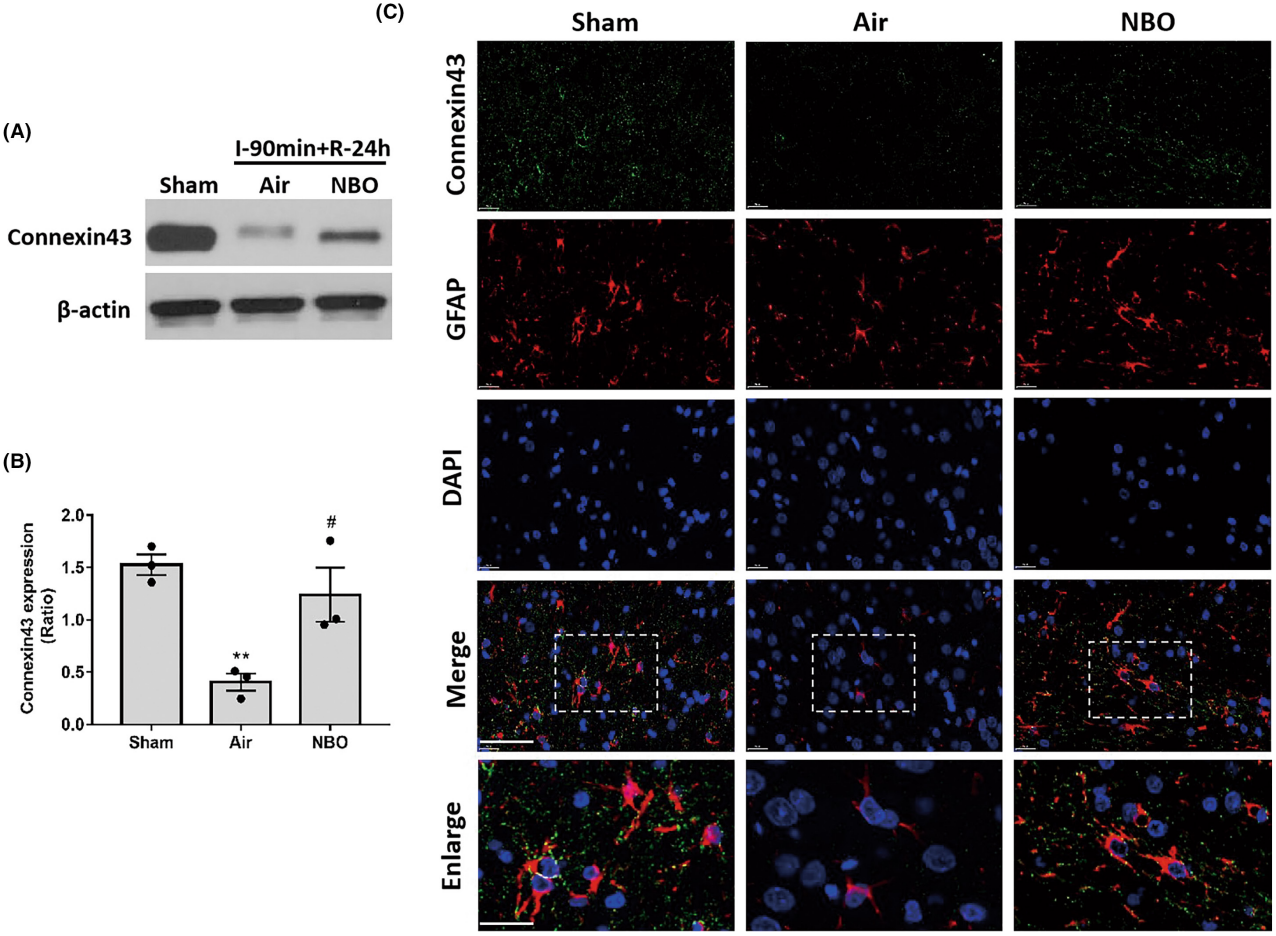
Normobaric hyperoxia treatment improved the expression of connexin43 in astrocytes after 90‐min ischemia/24‐h reperfusion. The level of connexin43 was measured using western blotting and immunohistochemistry staining after 90 min ischemia/24 h reperfusion. (A) Representative bands of western blotting. (B) Statistical analysis of connexin43 expression. Data are presented as the mean ± SEM. ^**^ *p* < 0.01, versus the sham group; ^#^ *p* < 0.05, versus the air group. (C) The co‐staining images of connexin43 (green) and anti‐glial fibrillary acidic protein (red) showed the expression of connexin43 in astrocytes with or without NBO treatment. The nucleus was stained with DAPI (blue). The images in white‐dashed boxes are enlarged. Scale bar: 50 μm (merge); 20 μm (enlarged). All data represent at least three independent experiments (three animals in each group)

To investigate the mechanism of NBO in the intercellular communication of astrocytes, we measured the levels of connexin43 in ischemic tissues after 90‐min ischemia plus 24‐h reperfusion using western blotting (Figure 3A). Statistical analysis showed that ischemia/reperfusion significantly reduced connexin43 expressions in ischemic tissues when compared with the sham rats. NBO alleviated the decrease of connexin43 during cerebral ischemia (Figure 3B). To demonstrate the effects of NBO treatment on connexin43 in astrocytes, immunohistochemistry staining (Figure 3C) was performed with co‐staining of connexin43 and GFAP (an astrocyte‐specific marker). The connexin43 signals were decreased in ischemic tissues compared with the normal group, suggesting that ischemia/reperfusion downregulated the expression of connexin43 in astrocytes. In contrast, NBO treatment increased the expression of connexin43 in GFAP‐positive cells. Together, these results suggested that NBO treatment significantly rescued the ischemia/reperfusion‐induced downregulation of connexin43 in astrocytes during acute cerebral ischemia.

### 3.3 NBO alleviates ischemia‐induced cytochrome c release via the GPX4‐connexin43 pathway

To further investigate the role of GPX4 and connexin43 in NBO‐induced protection, rats were pretreated with a connexin43 inhibitor (Gap27) or GPX4 activator (Ferrostatin‐1) to regulate the GPX4 pathway. Because CytC release is crucial for mitochondria‐dependent cell death, the mitochondrial and cytosolic fractions were isolated from ischemic tissues after cerebral ischemia/reperfusion.

**FIGURE 4:**
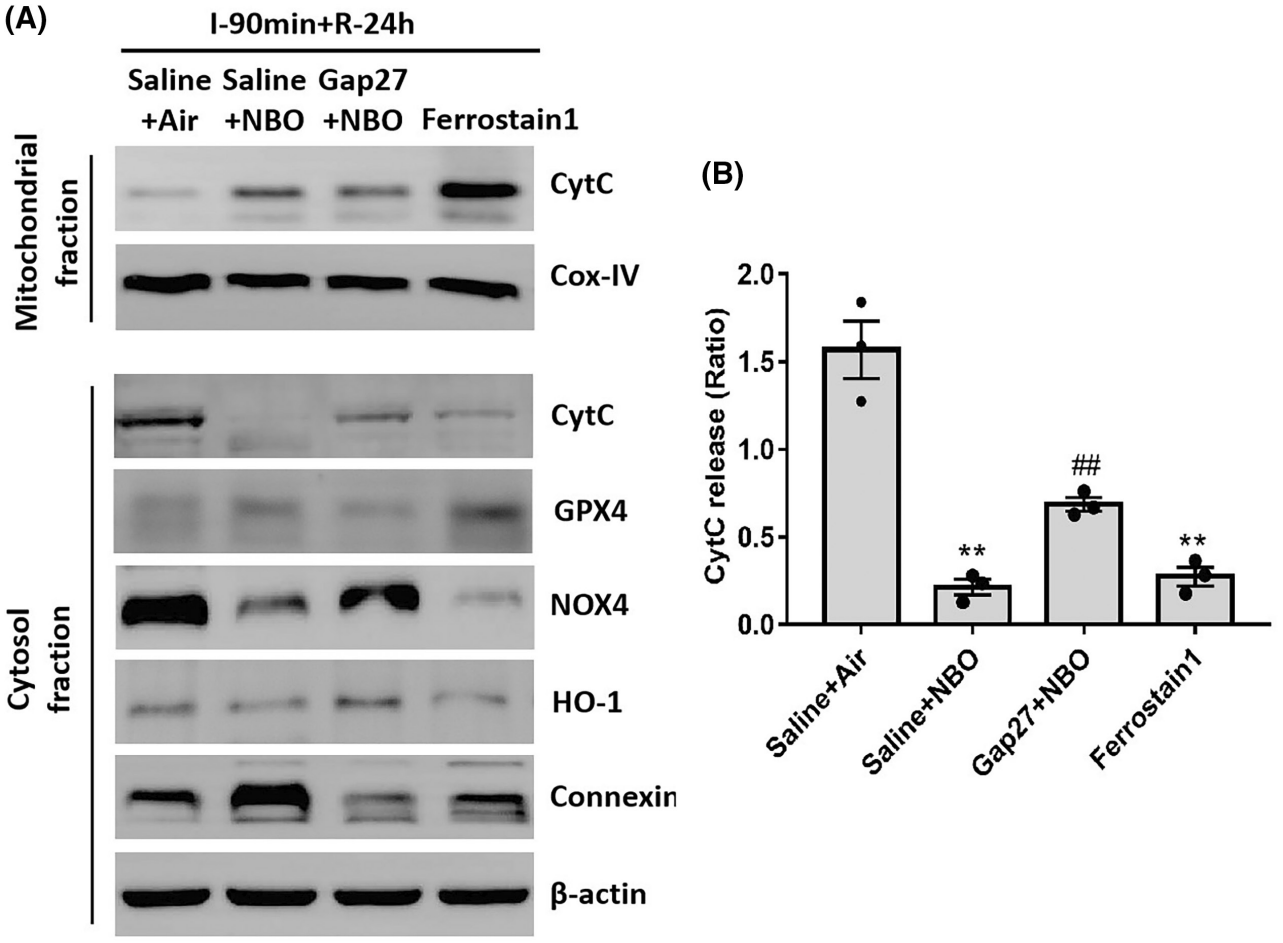
Normobaric hyperoxia treatment attenuated ischemia‐induced cytochrome c release via the GPX4‐connexin43 pathway. To investigate the role of the GPX4 or connexin43 pathway in NBO‐induced protection, rats were pretreated with connexin43 inhibitor (Gap27) or GPX4 activator (Ferrostatin‐1) by tail vein injections. The mitochondrial and cytosolic fractions were isolated from ischemic tissues to measure the levels of CytC in mitochondrial and cytosol fractions. (A) The level of CytC in isolated mitochondrial and cytosolic fractions. The level of Cox‐IV was shown as the loading control in the mitochondria fraction. The levels of NOX4, GPX4, HO‐1, and connexin43 in each group are also illustrated, showing the successful regulation of Gap27 and Ferrostatin‐1. (B) Statistical analysis of CytC release. Data are presented as the mean ± SEM. ^**^ *p* < 0.01, versus the saline + air group; ^##^ *p* < 0.01, versus the saline + NBO group. All data represent at least three independent experiments (three animals in each group)

As shown in Figure 4A, there was a low level of CytC in isolated mitochondria from ischemia/reperfusion rats. NBO treatment rescued the ischemia‐induced CytC reduction in mitochondria, which was partially blocked by inhibiting connexin43 with Gap27. Moreover, activating GPX4 using Ferrostatin‐1 restored CytC expression in mitochondria. The level of Cox‐IV, shown as the loading control, was constant in the mitochondria fraction.

The level of CytC in the cytosolic fraction was also measured to confirm CytC release in response to ischemia/reperfusion with or without NBO treatment. A high level of CytC in the cytosolic fraction was observed in cerebral ischemic rats. NBO treatment attenuated ischemia‐induced CytC release into the cytosolic fraction, which was significantly blocked by inhibiting connexin43 with Gap27. Increasing GPX4 activation using Ferrostatin‐1 significantly decreased CytC expression in the cytosolic fraction. The levels of NOX4, GPX4, HO‐1, and connexin43 in each group are shown in Figure 4A, indicating successful regulations by Gap27 and Ferrostatin‐1. The level of CytC release, which was expressed as the ratio in the cytosolic and mitochondria fractions, is shown in Figure 4B.

Overall, these results suggested that NBO treatment reduced ischemia‐induced mitochondrial damage, by inhibiting oxidative stress and astrocytic communications via the GPX4‐connexin43 pathway.

### 3.4 The GPX4‐connexin43 pathway participates in NBO‐induced neuroprotection after cerebral ischemia

**FIGURE 5:**
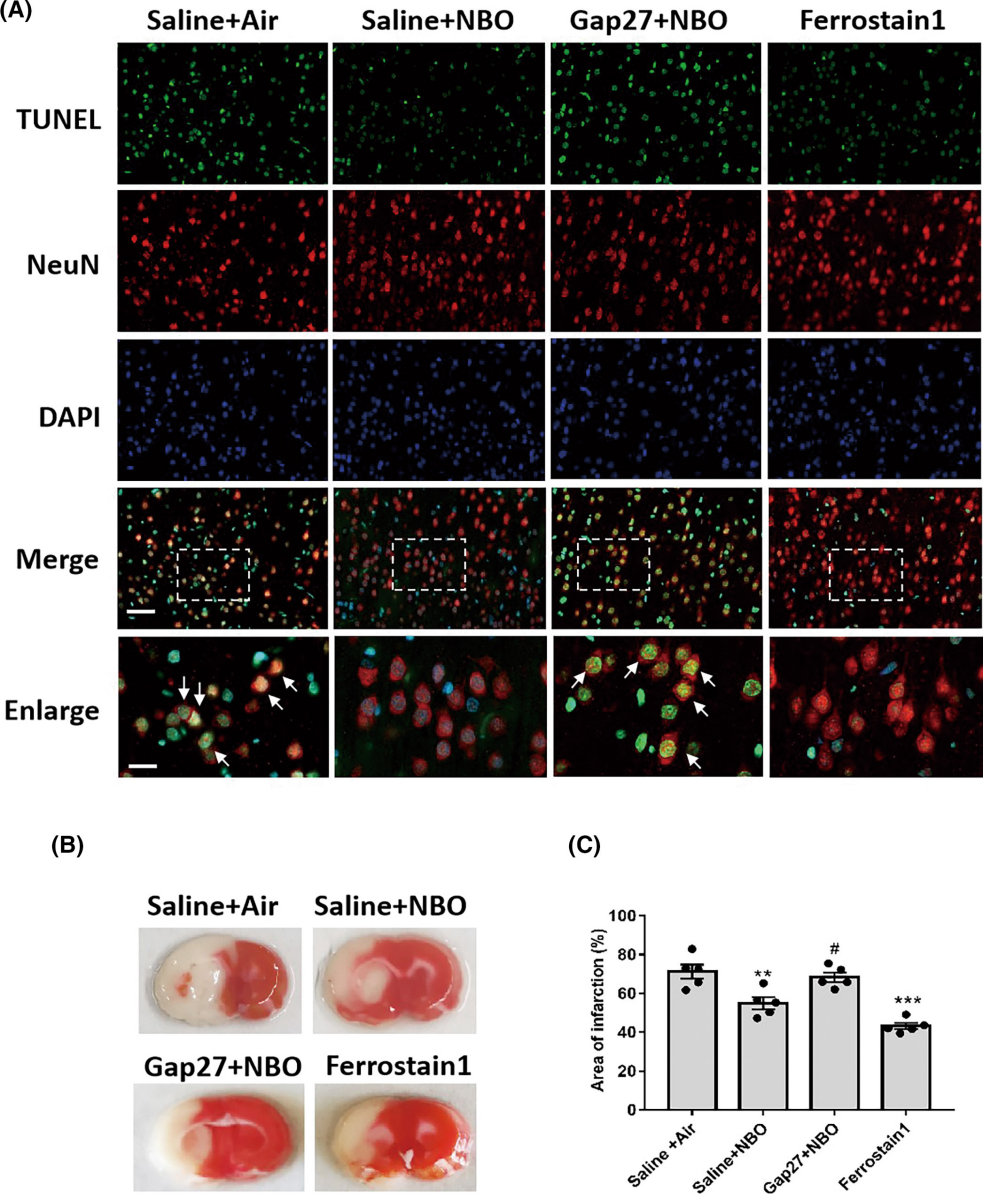
The GPX4‐connexin43 pathway participated in the normobaric hyperoxia (NBO)‐induced infarction reduction after cerebral ischemia. (A) TUNEL co‐staining (specific marker for apoptotic cells, colored green) and NeuN (specific marker for neurons, colored red). The nucleus was stained with DAPI (blue). The images in white‐dashed boxes were enlarged. Arrows refer to the TUNEL+/NeuN+ cells (neuronal cell death). Scale bar: 50 μm (merge); 20 μm (enlarged). (B) Typical TTC staining after 90‐min ischemia/24‐h reperfusion. (C) Statistical analysis. Data are presented as the mean ± SEM. ^**^ *p* < 0.01, ^***^ *p* < 0.001 versus the saline + air group; ^#^ *p* < 0.05, versus the saline + NBO group. All data represent at least five animals in each group

We further investigated whether the GPX4‐connexin43 pathway was responsible for NBO‐induced neuroprotection during ischemic stroke. To investigate the effects of the GPX4‐connexin43 pathway on neuronal survival/death, the neuronal cell death was measured using co‐staining of TUNEL (a specific marker for apoptotic cells, colored green) and NeuN (a specific marker for neurons, colored red). The co‐staining images showed that NBO treatment reduced the number of TUNEL+/NeuN+ cells (neuronal cell death, arrows) after cerebral ischemia/reperfusion, which was blocked by inhibiting connexin43 with Gap27. Increasing GPX4 activation using Ferrostatin‐1 decreased neuronal cell death after cerebral ischemia/reperfusion (Figure 5A).

TTC staining was measured to show the role of the GPX4‐connexin43 pathway in NBO‐induced neuroprotection during ischemic stroke (Figure 5B). Statistical analyses indicated that NBO significantly decreased the infarction volume when compared with control rats undergoing 90 min ischemia/24 h reperfusion. Inhibiting connexin43 with Gap27 partially abolished the neuroprotection of NBO. In contrast, Ferrostatin‐1 pretreatment decreased the ischemia‐induced infarction volume, which was similar to the results seen during NBO‐induced neuroprotection (Figure 5C).

**FIGURE 6:**
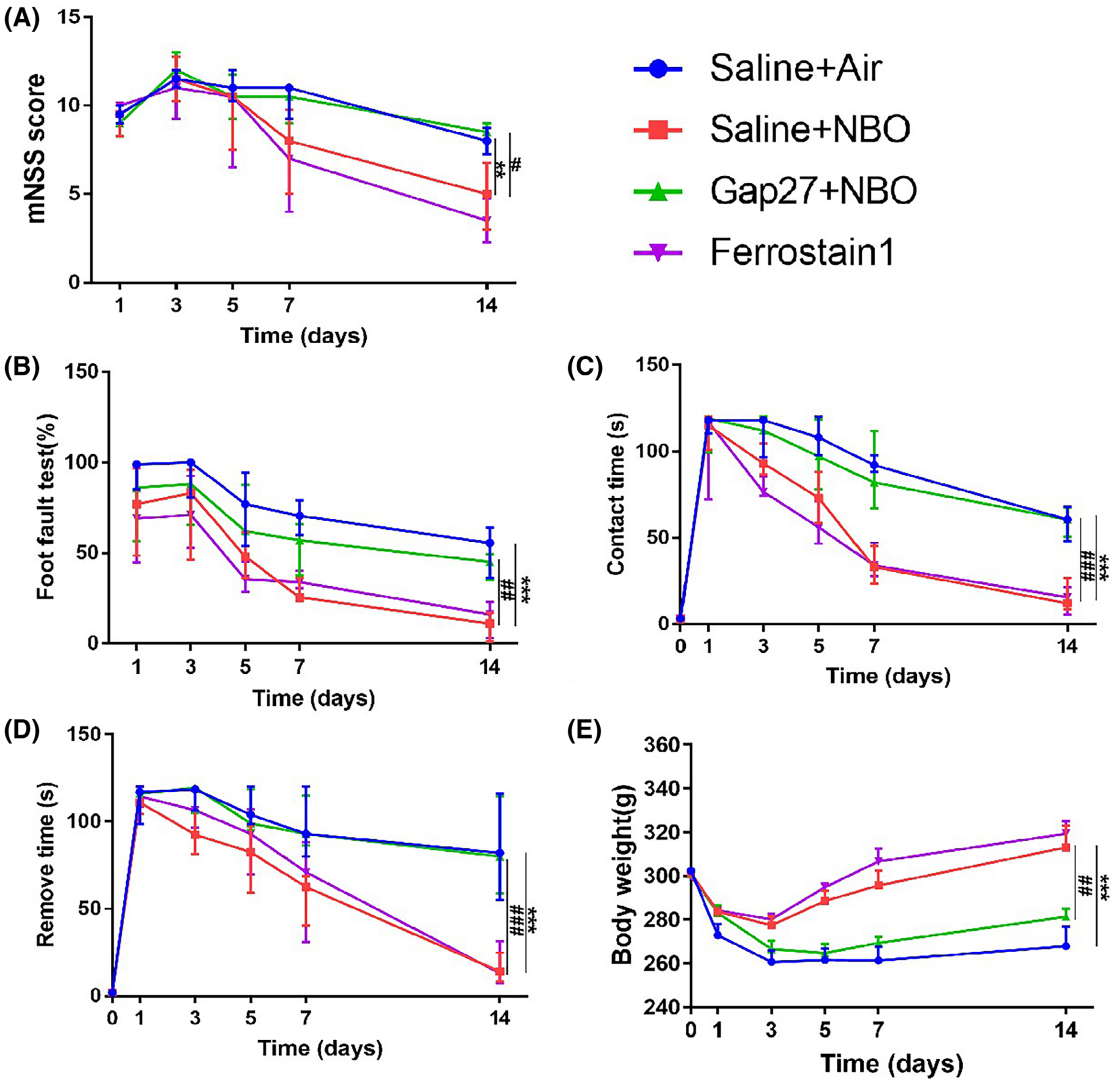
The GPX4‐connexin43 pathway participated in normobaric hyperoxia (NBO)‐induced neuroprotection after cerebral ischemia. The neurological functions and body weight were evaluated at 1, 3, 5, 7 and 14 days post ischemia/reperfusion. (A) The mNSS scores. (B) The foot‐fault test. (C) The contact time of the adhesive removal test. (D) The removal time of the adhesive removal test. (E) Body weight. Data are presented as median (interquartile range, A–D) or as the mean ± SEM (E). ^**^ *p* < 0.01, ^***^ *p* < 0.001 versus the saline + air group; ^#^ *p* < 0.05, ^##^ *p* < 0.01, ^###^ *p* < 0.001 versus the saline + NBO group. All data represent at least eight animals in each group

The prognosis within 14 days post ischemia/reperfusion was further evaluated, including neurological functions (mNSS scores, foot‐fault test, and the adhesive removal test; Figure 6A–D) and body weight (Figure 6E). NBO treatment significantly improved the prognosis at 14 days post cerebral ischemia, which was partially reversed by inhibiting connexin43. Blocking NOX4 activation with Ferrostatin‐1 decreased ischemia‐induced neurological deficits and poor prognoses, which was similar to the effect of NBO on the prognoses. Together, these results suggested that NBO suppressed oxidative stress in astrocytes and improved prognoses via the GPX4 and connexin43 pathways.

## 4 DISCUSSION

During recanalization, it is crucial to decrease the impact of ischemia and stabilize brain tissue, while waiting for vascular recanalization as early as possible.^24^ However, during non‐recanalization, most therapeutic drugs are unable to reach the ischemic area to exert their effects. Oxygen can easily diffuse to ischemic regions to enhance the oxygen supply for hypoxic tissues, so NBO is considered an ideal protective therapy for cerebral ischemia.^6^ Recent clinical studies further demonstrated that NBO significantly improves clinical outcomes in ischemic stroke patients.^8^

The present study investigated the role of astrocytes during the early phase in NBO treatment against cerebral ischemia/reperfusion. NBO treatment retained the homeostasis of oxidoreductases (GPX4/NOX4) and the level of connexin43 in astrocytes. NBO treatment attenuated ischemia‐induced CytC release from mitochondria, which was blocked by inhibiting the connexin43 pathway in astrocytes.

Recent studies indicated that astrocytes play important roles in supporting neuronal functions and survival during cerebral ischemia.^10^, ^11^ Connexin43, the predominant member of the connexin family in astrocytes, has been shown to play a role in cerebral ischemia, where it is responsible for cell–cell coupling and communication.^13^ Because previous studies showed that NBO was able to maintain a normal pO_2_ in ischemia‐affected tissues,^7^, ^25^ the present study focused on astrocytes in ischemia‐affected tissues. The results showed that acute obstruction of cerebral blood flow led to a rapid reduction of connexin43 expression in astrocytes in the ischemic‐affected areas during the early phase of ischemia, which was reversed by NBO treatment before recanalization. This suggested that NBO treatment improved the activity of astrocytes in the ischemic region and retained connections and/or communications close to normal conditions.

Redox homeostasis is required to keep the balance between the activation of oxidases and antioxidases under physiological conditions.^26^ The GPX4, an important antioxidase in suppressing lipid peroxidation, has been shown to play a key role in inhibiting ferroptosis.^27^ Recent studies showed that NOX4, oxygen‐ and NADPH‐dependent oxidoreductases, was acutely regulated by cellular pO_2_, and served as an O_2_ sensor in response to H_2_O_2_ signaling.^28^, ^29^ The present study showed that NBO maintained the balance of redox (elevating GPX4 and suppressing NOX4 expression), attenuated oxidase activity downstream (HO‐1) in ischemia‐affected astrocytes, and eliminated mitochondrial damage in the form of cytochrome c release into cytosolic fractions. Inhibiting connexin43 using Gap27 eliminated NBO‐induced redox homeostasis in astrocytes, while activating GPX4 using Ferrostatin‐1 (as a positive control of NBO) showed effects similar to NBO treatment. Overall, these data suggested that connexin43 and GPX4 contributed to the mechanism of NBO‐induced redox homeostasis.

Oxygen therapy has been shown to increase the risks of oxidative stress, leading to inflammation and tissue damage. The risks of oxidative stress induced by oxygen therapy may be largely dependent on the duration of prolonged exposure.^30^, ^31^, ^32^ In this study, we showed that a short duration of NBO reduced mitochondrial damages by maintaining redox homeostasis in astrocytes, rather than increasing oxidative stress. A previous study showed that NBO did not increase oxidative stress within the acute treatment window.^33^ NBO treatment resulted in relatively healthy astrocytes with a balanced redox system, which maintained mitochondrial functions and decreased ischemic injury in brain tissues. These characteristics of NBO (relatively safe, well‐tolerated, with few side effects) make it a potential treatment to “buy time” for recanalization therapies.

It is worth noting that sexual dimorphism exists in brain vessels, cerebral blood flow, brain metabolism, and stroke outcomes.^34^, ^35^, ^36^, ^37^ In the present study, male animals were used to test our mechanistic hypothesis. The neuroprotective effects of NBO on female animals will be investigated in the future studies.

In summary, this study showed that NBO may play a neuroprotective role by reducing oxidative stress and maintaining the level of connexin43 in astrocytes. This study suggested a possible NBO treatment for clinical applications in patients with acute ischemic stroke.

## AUTHOR CONTRIBUTIONS

ZQ, SY, and XJ made the study design; ZQ and SY did experiments and wrote the manuscript. KL and XJ revised the manuscript.

## CONFLICT OF INTEREST

None.

## Supporting information




**Appendix S1** Supplementary Information

## Data Availability

The data that support the findings of this study are available from the corresponding author upon reasonable request.
