# Supplementary material for: Normobaric hyperoxia plays a neuroprotective role after cerebral ischemia by maintaining the redox homeostasis and the level of connexin43 in astrocytes
Source: CNS Neurosci Ther. 2022 Jun 14;28(10):1509–18. doi: 10.1111/cns.13875 (PMC9437237; doi:10.1111/cns.13875)

Full unedited gel for Figure 2A

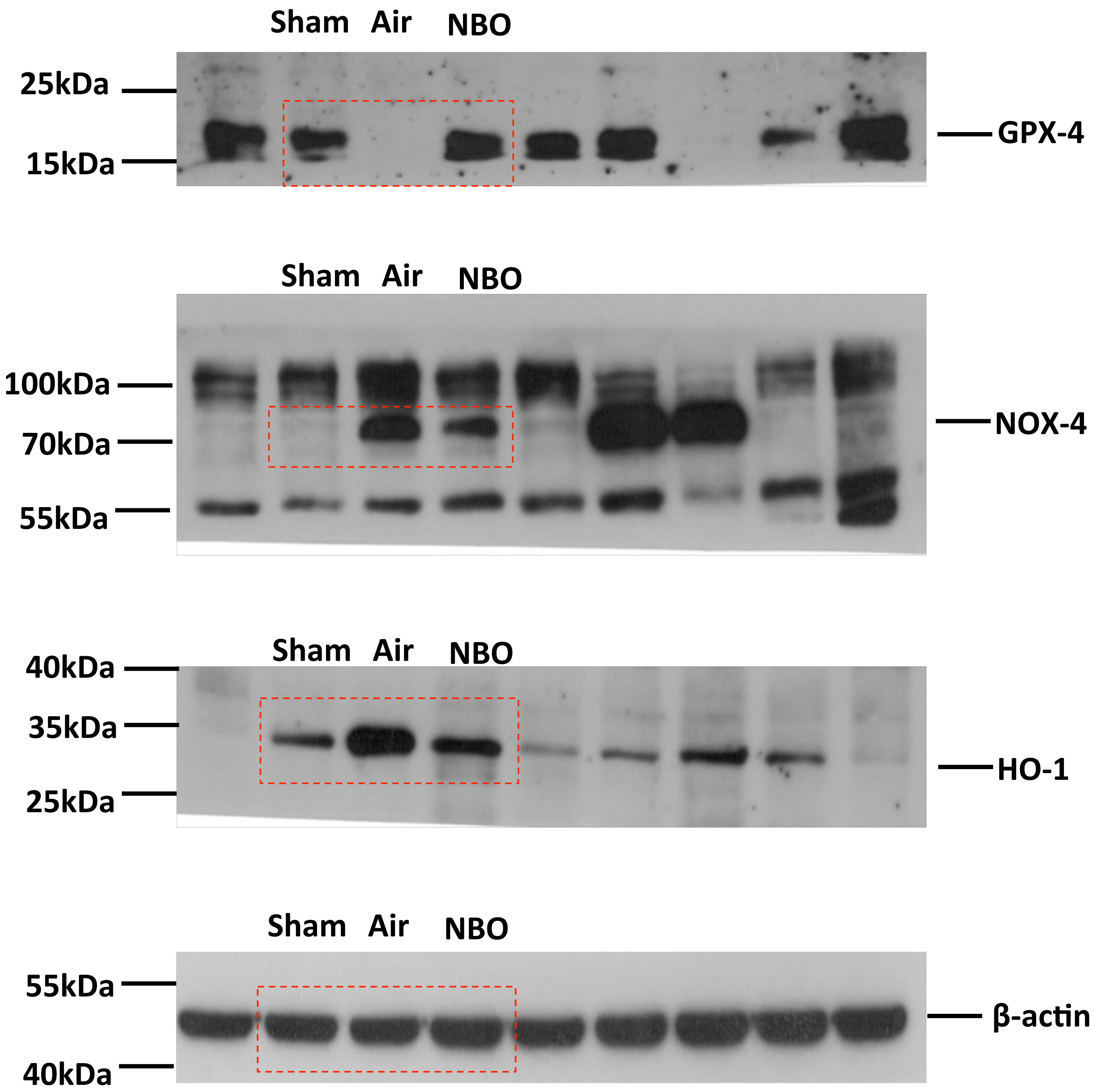

Full unedited gel for Figure 3A

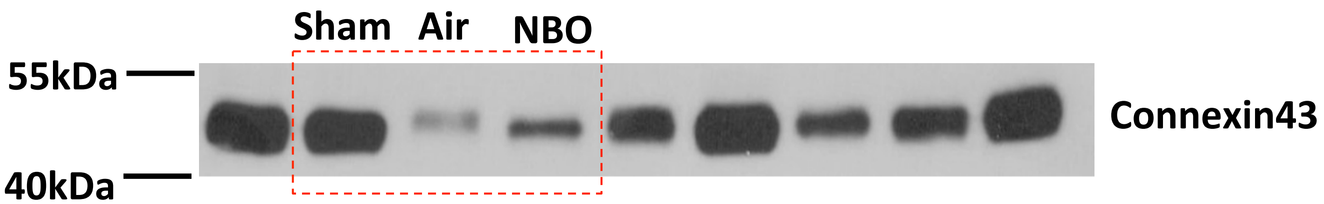

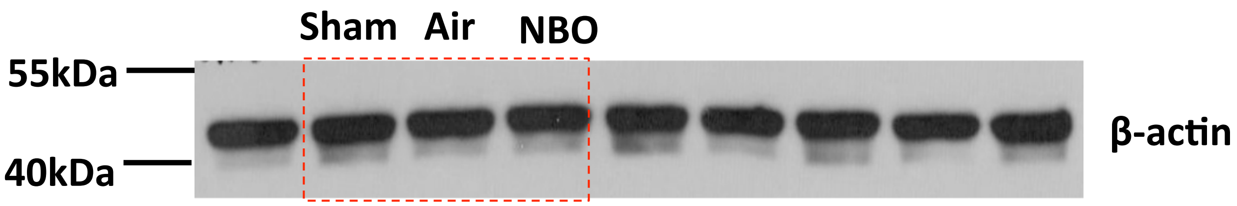

Full unedited gel for Figure 4A

Mitochondrial fraction

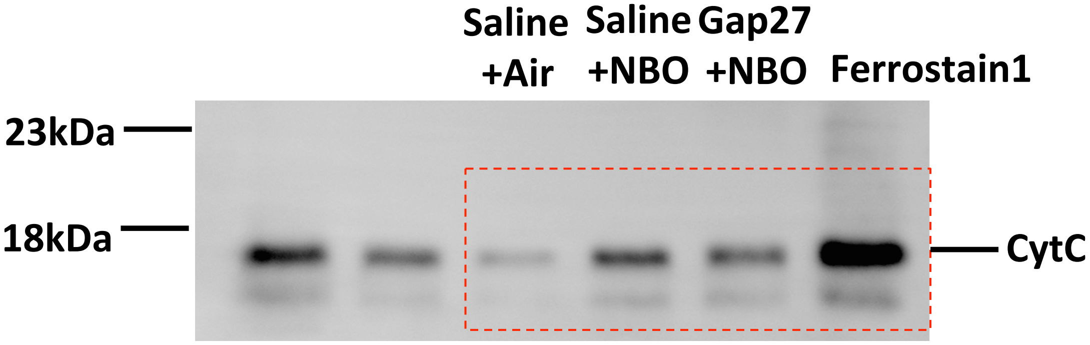

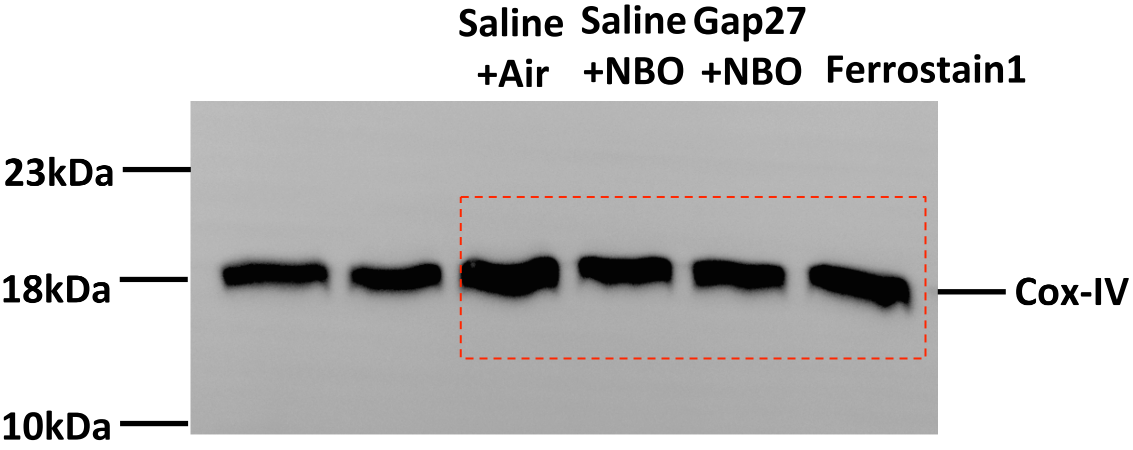

Full unedited gel for Figure 4A

Cytosol fraction

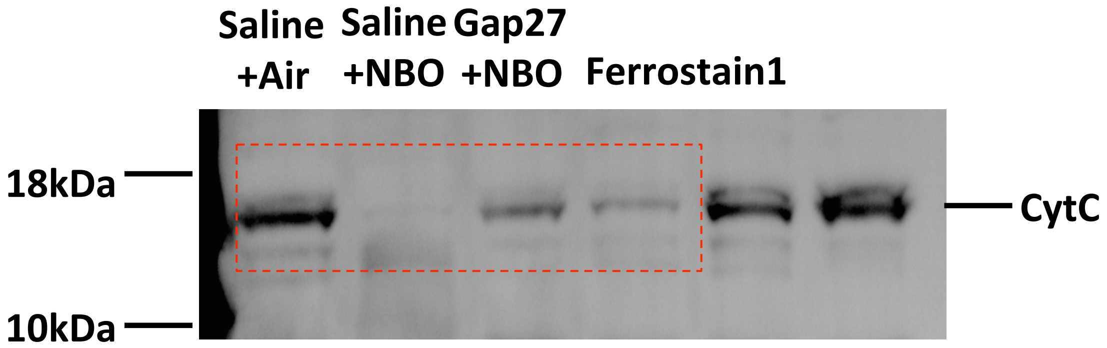

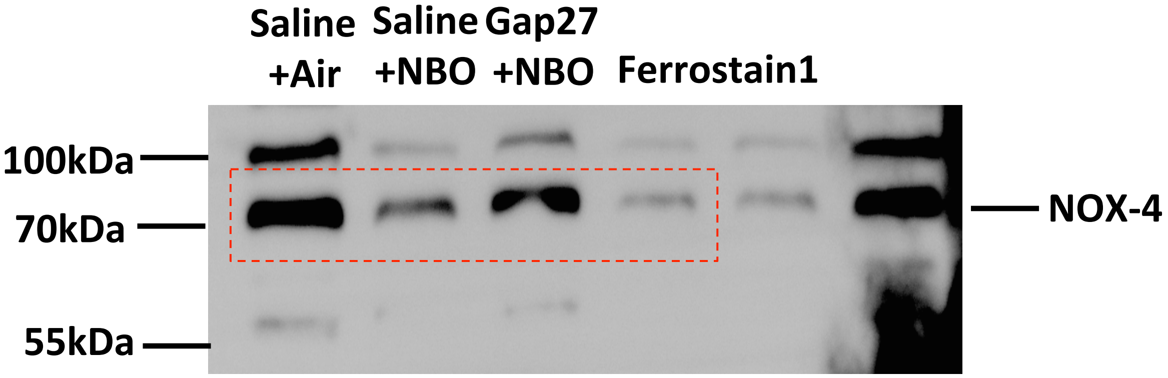

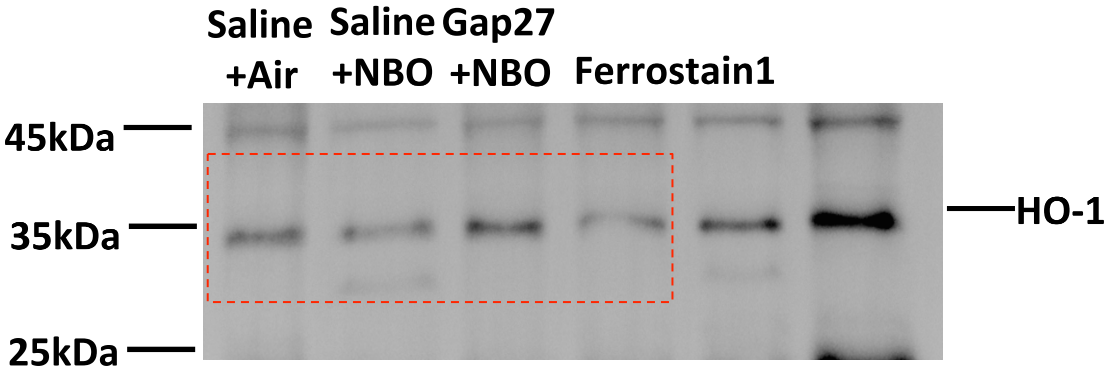

Full unedited gel for Figure 4A

Cytosol fraction

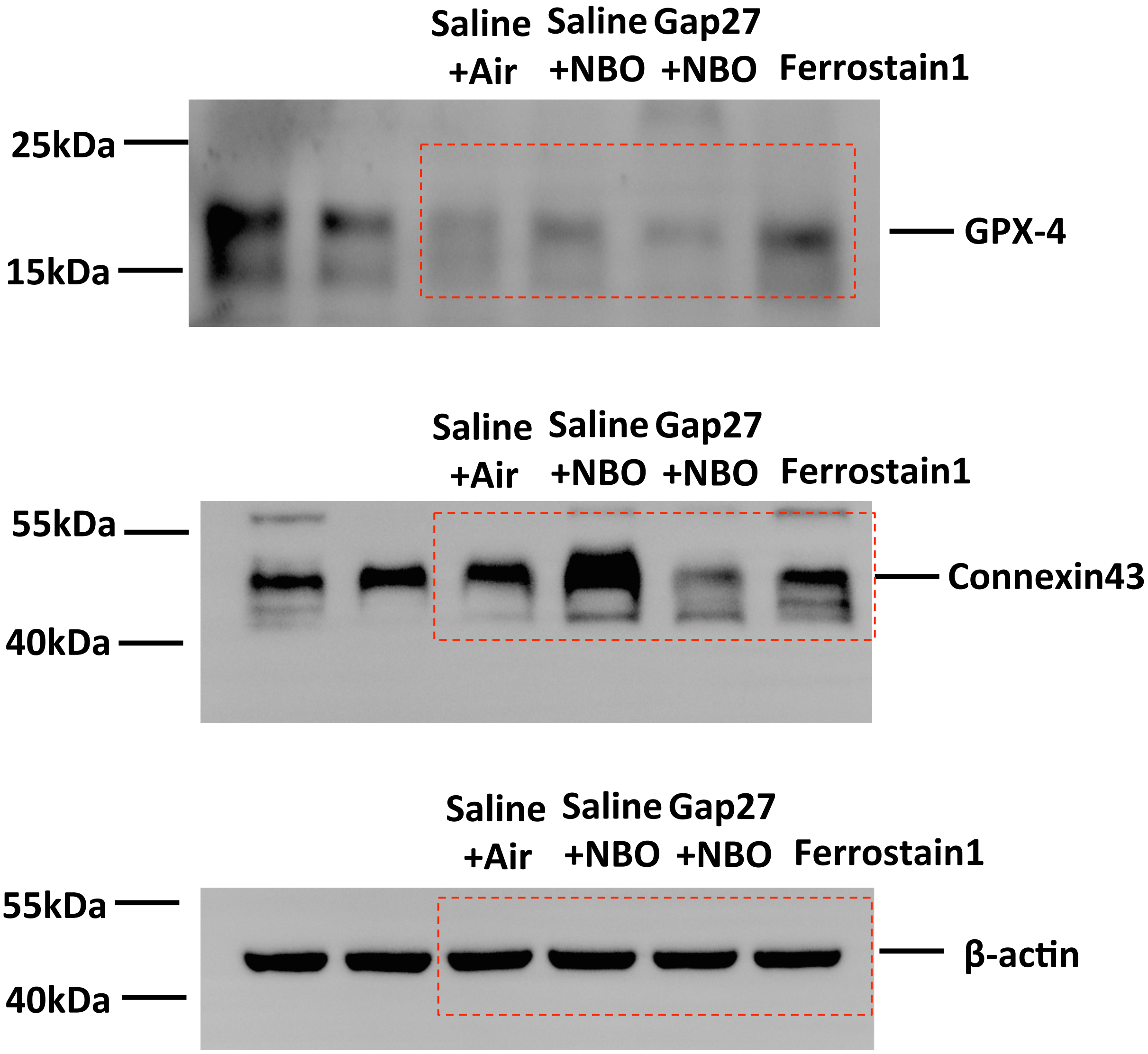

Supplement: Supplementary file 1 — Appendix S1 Supplementary Information [file CNS-28-1509-s001.pdf]
